# Supplementary material for: Cardiovascular risk factors and its patterns of change between 4 and 8 years of age in the INMA-Asturias cohort
Source: PLoS One. 2023 Apr 12;18(4):e0283799. doi: 10.1371/journal.pone.0283799 (PMC10096221; doi:10.1371/journal.pone.0283799)
Supplement: S1 File — (DOCX) [file pone.0283799.s002.docx]

**Supplementary material**

**
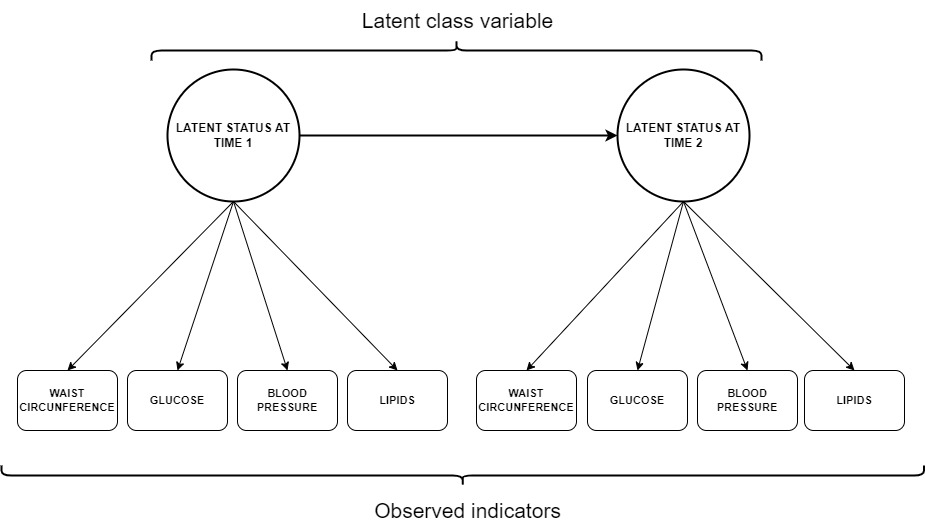
**

**S1 Figure.**  **Graphical representation of LTA model.**

**Supplementary material S1:** **Description of the process for specifying, estimating, and selecting the final LTA model.**

In the first step, models from two to five latent statuses were estimated. To be able to interpret the latent status in a meaningful way, good homogeneity and latent class separation were considered to select the number of latent statuses in the model. Entropy was also used to select the best model as a measure of classification uncertainty (this measure can range from 0 to 1, with higher values representing a better fit, and > 0.7 is considered acceptable) (28). Akaike information criterion and Bayesian information criterion values were considered. The prevalence of the different latent statuses was not much lower than 10%, to avoid detecting excessively minority patterns.

The second and third steps involved testing the hypothesis of longitudinal measurement invariance and the hypothesis of change between time points invariance, respectively, using the likelihood ratio test. In the longitudinal measurement invariance hypothesis, the identified latent status is the same at 4 and 8 years of age. If this hypothesis is accepted, the latent status at 4 and 8 years is forced to be the same, which constrains the item-response probabilities to be the same at both time points. In contrast, if this hypothesis is rejected, the latent status can be assumed to be different at 4 and 8 years, and item-response probabilities can be freely estimated. The longitudinal measurement invariance hypothesis should usually be assumed to make the model easier to interpret because fewer parameters need to be estimated under this assumption. However, this study was exploratory in nature, and we did not assume an a priori hypothesis on the behavior of the latent status in children. Therefore, this hypothesis was tested. In the change between time points invariance hypothesis, children who are in a latent status at 4 years of age will be in the same latent status at 8 years of age, without the possibility of change. If this hypothesis is accepted, the transition probabilities are constrained to be equal to 0, and if it is rejected, the transition probabilities are freely estimated.

After this process, the three latent status model was selected. Although this model did not have the best information criterion values (Akaike information criterion and Bayesian information criterion) and the highest entropy, it had good homogeneity, latent class separation, and interpretability. A summary of information on the relative model fit for selecting the number of latent statuses is shown in Supplementary Table S2.

The hypothesis of longitudinal measurement invariance was rejected (*p* = 0.009, see S2 Table). Therefore, although constraining the item-response probabilities to be equal across times would make interpreting the model easier, it would not be a reasonable assumption and would not capture the underlying structure of the data. On the basis that the latent status was different at the two time points, the hypothesis of change between time points invariance was also rejected because children who were in a latent status at T0 inevitably changed to another latent status at T1. Therefore, transition probabilities between T0 and T1 were different from zero.

**S1 Table. Number and percentage of children with zero, one, two, three or four risk factors at the monitoring level**.

| Nº of risk factors at the monitoring level | Disorders | T0 | | T1 | |
| --- | --- | --- | --- | --- | --- |
|  |  | N | % | N | % |
| 0 | WC- G- BP- LIP- | 76 | 29.0% | 92 | 36.9% |
| 1 | WC+ G- BP- LIP- | 25 | 9.5% | 18 | 7.2% |
| 1 | WC- G+ BP- LIP- | 17 | 6.5% | 5 | 2.0% |
| 1 | WC- G- BP+ LIP- | 32 | 12.2% | 51 | 20.5% |
| 1 | WC- G- BP- LIP+ | 35 | 13.4% | 31 | 12.4% |
| 2 | WC+ G+ BP- LIP- | 6 | 2.3% | 2 | 0.8% |
| 2 | WC+ G- BP+ LIP- | 8 | 3.1% | 16 | 6.4% |
| 2 | WC+ G- BP- LIP+ | 18 | 6.9% | 10 | 4.0% |
| 2 | WC- G+ BP+ LIP- | 7 | 2.7% | 4 | 1.6% |
| 2 | WC- G+ BP- LIP+ | 8 | 3.1% | 2 | 0.8% |
| 2 | WC- G- BP+ LIP+ | 8 | 3.1% | 7 | 2.8% |
| 3 | WC+ G+ BP+ LIP- | 3 | 1.1% | 0 | 0.0% |
| 3 | WC- G+ BP+ LIP+ | 3 | 1.1% | 1 | 0.4% |
| 3 | WC+ G- BP+ LIP+ | 7 | 2.7% | 8 | 3.2% |
| 3 | WC+ G+ BP- LIP+ | 8 | 3.1% | 1 | 0.4% |
| 4 | WC+ G+ BP+ LIP+ | 1 | 0.4% | 1 | 0.4% |

WC+: Waist circumference at the monitoring level. WC-: Waist circumference at the normal level.

G+: Blood glucose at the monitoring level. G-: Blood glucose at the normal level.

BP+: Blood pressure at the monitoring level. BP-: Blood pressure at the normal level.

Lip+: Lipid levels at the monitoring level. Lip-: Lipid levels at the normal level.

**S2 Table. Summary of information for selecting the number of latent status and fit statistics for test the hypothesis of measurement invariance.**

| Measurement invariance | Number of latent status | Number of paremeters estimated | G^2^ | df | AIC | BIC | LL | Entropy | p-value* |
| --- | --- | --- | --- | --- | --- | --- | --- | --- | --- |
| Yes | 2 | 11 | 201.7 | 241 | 2477.9 | 2519.7 | -1227.9 | 0.791 |  |
| No | 2 | 19 | 168.1 | 234 | 2457.6 | 2529.9 | -1209.8 | 0.800 | <0.001 |
| Yes | 3 | 20 | 174.3 | 233 | 2466.2 | 2542.3 | -1213.1 | 0.688 |  |
| No | 3 | 32 | 145.9 | 221 | 2460.7 | 2582.5 | -1198.4 | 0.767 | <0.001 |
| Yes | 4 | 31 | 150.9 | 222 | 2465.1 | 2583.0 | -1201.5 | 0.842 |  |
| No | 4 | 47 | 127.9 | 206 | 2470.9 | 2649.8 | -1188.5 | 0.855 | 0.009 |
| Yes | 5 | 44 | 130.6 | 209 | 2470.9 | 2638.3 | -1191.5 | 0.819 |  |
| No | 5 | 64 | 108.5 | 189 | 2485.4 | 2728.9 | -1178.7 | 0.840 | 0.19 |

*p-value obtained from the chi-square difference test based on loglikelihood values for testing the hypothesis of measurement invariance across times. G^2^, likelihood-ratio statistic. df, degrees of freedom. AIC, Akaike information criterion. BIC, Bayesian information criterion. LL, loglikelohood value.
