# Supplementary material for: Cardiovascular risk factors and its patterns of change between 4 and 8 years of age in the INMA-Asturias cohort
Source: PLoS One. 2023 Apr 12;18(4):e0283799. doi: 10.1371/journal.pone.0283799 (PMC10096221; doi:10.1371/journal.pone.0283799)
Supplement: Attachment — Submitted filename: Review comments on Manuscript Number.doc [file pone.0283799.s003.doc]

**Review comments on Manuscript Number: PONE-D-22-28738. Entitled "Cardiovascular risk factors and its patterns of change between 4 and 8 years of age in the INMA-Asturias Cohort"**

Overall, the idea of research is very interesting to be studied nowadays and paper is coherently developed. However, there are some comments and suggestions.

**Title**

- Well structured

**Abstract**

- It is recommended to write the abstract in organized sections (background, aim, methods, results and conclusion)
- Conclusion is rephrasing of the results. It is recommended to provide your conclusion and comment on the findings
- Keywords: write it in alphabetical order

**Introduction**

- Well structured

**Materials and methods**

- Well structured

**Statistical analysis**

- Well structured. However, too many details are written you may need to concise this section.

**Discussion**

- Well structured
